# Histidine is essential for growth of *Komagataella phaffii* cultured in YPA medium

**DOI:** 10.1002/2211-5463.13408

**Published:** 2022-05-12

**Authors:** Aditi Gupta, Pundi N. Rangarajan

**Affiliations:** ^1^ 29120 Department of Biochemistry Indian Institute of Science Bangalore India

**Keywords:** acetate metabolism, F1F0 ATPase, histidine, histidine‐responsive genes, *Komagataella phaffii*

## Abstract

*Komagataella phaffii* (*a.k.a. Pichia pastoris*) requires histidine for optimal growth when cultured in a medium containing yeast extract, peptone (YP), and acetate (YPA). We demonstrate that *HIS4*‐deficient, *K. phaffii* strain *GS115* exhibits a growth defect on YP‐media containing acetate, but not on other carbon sources. *K. phaffii X33*, a prototroph, grows better than *K. phaffii GS115* (*his4*), a histidine auxotroph in YPA. Normal growth of *GS115* is restored either by the expression of *HIS4* or by culturing in YPA containing ≥0.6 mM histidine. In the presence of histidine, expression of several genes is altered, including those encoding key subunits of mitochondrial ATP synthase, transporters of amino acids and nutrients, as well as biosynthetic enzymes. Thus, histidine should be included as an essential component for optimal growth of *K*. *phaffii* histidine auxotrophs cultured in YPA.

## Graphical abstract

*Komagataella phaffii GS115* (*his4*), a histidine auxotroph grows poorly in a medium containing yeast extract, peptone, and acetate (YPA) compared to *X33*, a prototroph. Expression of *HIS4* in *GS115* or supplementation of YPA medium with histidine restores normal growth. Several histidine responsive genes were identified by RNA‐seq such as those involved in ATP synthesis, uptake of amino acids, and amino acid biosynthesis.

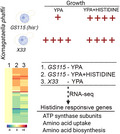

**Abbreviations**
HIS4: histidinol dehydrogenase
YNB: yeast nitrogen base
YP: medium containing yeast extract and peptone
YPA: YP + acetate
YPAH: YPA + histidine

The ability of the respiratory yeast *Komagataella phaffii* (*a.k.a. Pichia pastoris*) to metabolize methanol has been exploited successfully to develop alcohol oxidase 1 (*AOXI*) promoter‐based expression systems [1, 2]. Usually, recombinant strains of *K. phaffii* are cultured in media containing glucose or glycerol for achieving high cell density, and then transferred to methanol for induction. During the growth phase, metabolic intermediates in the form of yeast extract and peptone are often provided in the medium to enhance the growth rate further. *K. phaffii* can also grow on ethanol, and ethanol is used for high cell density fermentation [3]. However, acetate does not support robust growth and therefore is not used for high‐density fermentation [4]. Recent studies have highlighted the importance of acetate in the biomanufacturing of acetate‐derived compounds [5]. This has led researchers to tweak the acetate utilization and related processes in yeasts to achieve tolerance to higher acetate levels. For example, screening for acetate‐sensitivity kinases led to the identification of *HRK1,* a kinase involved in the regulation of plasma membrane H (+)‐ATPase Pma1 in *K. phaffii*. Overexpression of *HRK1* resulted in high growth in presence of 30 mM (~0.2%) acetate leading to a 55% increase in the product derived from acetyl Co‐A as compared to the wildtype strain [6]. However, there are still efforts to maximize the acetate utilization in *K. phaffii*.

Acetate, a weak acid, is the precursor of acetyl Co‐A and is found in abundance in nature. Only a few yeasts, such as *Saccharomyces cerevisiae* and *K. phaffii* are tolerant of acetate [6, 7]. Acetate utilization in yeasts begins with the internalization of acetate through the membrane transporters, followed by its ligation to Co‐A by acetyl Co‐A synthetases 1 and 2 (ACS1, ACS2) with ATP as the phosphate group donor. Acetyl Co‐A, thus formed, has many fates inside the cell. It can be channelized into the tricarboxylic acid cycle or the glyoxylate cycle, utilized for fatty acid biosynthesis or acetylation of proteins, including histones [5]. Acetate metabolism of *K. phaffii* is regulated by transcription factors such as Mxr1, Cat8‐1, and Cat8‐2 [3, 8].

For biomanufacturing of desired products, the gene(s) of interest is expressed in the host strain, using an expression vector harboring an appropriate selection marker. In *K. phaffii,* several auxotrophic strains have been developed for this purpose by exploiting the genes involved in the biosynthesis of histidine, methionine, arginine, adenine, uracil, lysine, tyrosine, proline, and phenylalanine such as *HIS4, MET2, ARG4, ADE1, URA3, LYS2, TYR1, PRO3,* and *PHA2,* respectively [9, 10, 11, 12]. Of these, the histidine auxotroph *GS115*, harboring a defective *his4* gene encoding histidinol dehydrogenase, is widely used for heterologous protein production [9].

In this study we demonstrate that wildtype *K. phaffii* strain (*X33*), but not histidine auxotroph (*GS115*), grows efficiently in a complex medium containing 1% yeast extract, 2% peptone (YP), and 2% acetate (YPA), suggesting a role for *HIS4‐*mediated histidine biosynthesis for adequate growth. Optimal growth of *GS115* can also be achieved by adding ≥0.6 mM histidine to YPA (YPAH). Histidine induces significant changes in the gene expression profile, as evident from high‐throughput genome‐wide RNA sequencing. Key histidine‐responsive genes identified in this study include those involved in ATP synthesis, transport of amino acids and nutrients, and biosynthetic pathways.

## Materials and methods

### Growth media and culture conditions

*K. phaffii* cells were maintained in nutrient‐rich YPD agarose plates (1% yeast extract, 2% peptone, and 2% dextrose). A single colony was grown overnight in YPD at 30°C in an orbital shaker at 180 rpm followed by washing with sterile distilled water (twice) and transferred to desired media containing YP (1%yeast extract, 2% peptone) and a carbon source (2%) such as dextrose (YPD), acetate (YPA), glycerol (YPG). Media were supplemented with L‐histidine (20–100 μg·mL^−1^), L‐methionine (20‐100 μg·mL^−1^) or L‐glutamate (20‐100 3, μg·mL^−1^) or ammonium sulfate (0.5%) when required. The minimal medium consisted of 0.67% yeast nitrogen base (YNB) with amino acids and an appropriate carbon source (2%). *Escherichia coli TOP10* was used for plasmid isolation. Bacterial and yeast transformations were done using the CaCl_2_ method and electroporation (Gene Pulsar, Bio‐Rad, Hercules, CA, USA), respectively. Yeast extract was procured from Thermo Fisher, Bangalore, India (#212750) or Himedia, Mumbai, India (#RM027). Peptone was purchased from Thermo Fisher (#211677) or Himedia (#RM001).

### Antibodies and other reagents

Mouse anti‐Myc antibodies were purchased from Merck Millipore (Bangalore, India). Restriction enzymes and T4 DNA ligase were purchased from New England Biolabs (Ipswich, MA, USA). DNA polymerases were purchased from GeNei (Bangalore, India). Oligonucleotides were purchased from Sigma‐Aldrich, India.

### Growth kinetics

A single colony grown till late log phase was pelleted and washed twice with autoclaved milliQ (Millipore, Bedford, MA, USA) water under sterile conditions, resuspended in water, and inoculated into specific media at an initial A_600_ of 0.07 and grown at 30 °C at 180 rpm in an orbital shaker. A_600_ was measured at regular intervals.

### Spot plate assay

Cells were grown in YPD till A_600_ = 2.0–4.0 and 1.0 O.D., and cells were suspended in 1 mL water for serial dilution preparation. One μl from each serial dilution was spotted on YNBD His^‐^ or His+ plates and incubated at 30 °C for 2–3 days.

### Western blotting

Cells were lysed using the glass‐bead lysis method. Proteins were quantified using Bradford reagent, resolved on SDS polyacrylamide gels, and transferred to PVDF membrane as described [4]. Blots were probed with primary and secondary antibodies using standard protocols.

### RNA‐sequencing and data analysis

Total RNA was isolated from *GS115* and *X33* cultured in YPA and *GS115* cultured in YPAH^5x^ in duplicate for 24 h by Qiagen (Chatsworth, CA, USA) RNeasy kit according to the manufacturer’s protocol. RNA Seq was performed using Illumina HiSeq at Clevergene (Bangalore, India). Differentially expressed genes were analyzed as described previously [4]. Genes with absolute log2‐fold change ≥2 and adjusted *P*‐value < 0.05 were considered significant. The expression profile of differentially expressed genes across the samples is presented in volcano plots and heatmaps. The three‐letter gene names were obtained by submitting the Uniref100 IDs to UniProtKB. However, in this process there was some data loss, as all proteins could not be mapped. The transcriptome datasets generated in the current study are available at the NCBI with the accession number GSE174633.

### Generation of *GS115‐HIS4*

The *pIB3* vector harboring *HIS4* as a selection marker (Addgene, plasmid # 25452) [13] was linearized with SalI, transformed into *GS115* for integration into the *HIS4* locus. Colonies were selected on YNBD His^‐^ plates.

### Generation of *GS115‐HIS4^Myc^*

*pGAP‐HIS4^Myc^* was constructed by cloning the *HIS4* into *pGAPZA* (Thermo Fisher, Bangalore, India) downstream of the *GAPDH* promoter in‐frame with the vector‐encoded c‐Myc epitope. *HIS4* was amplified from *pIB3* using the forward primer (1–23 bp of *HIS4*) 5′‐CCGGAATTCATGACATTTCCCTTGCTACCTGC‐3′ (F) and the reverse primer (complementary to C‐2508–2529 bp of *HIS4*) 5′ATAAGAATGCGGCCGCTAATAAGTCCCAGTTTCTCCATACGA‐3′ (R). EcoRI and NotI sites are underlined. The PCR product was digested with *EcoRI* and *NotI,* and cloned into pGAPZA to obtain *pGAP‐HIS4,* which was transformed into *P. pastoris GS115*, a histidine auxotroph. Cells were plated onto YPD + Zeocin plates and Zeo colonies were selected. Expression of Myc*‐*tagged His4 (His4^Myc^) was confirmed by western blotting using anti‐Myc epitope antibodies and histidine prototrophy was confirmed by plating on YNBD (His^‐^) plates.

### Generation of *GS115‐H670A^Myc^ GS115‐H737A^Myc^*

The specific point mutations in the *HIS4* were generated by site‐directed mutagenesis. *pGAP‐HIS4* was used as a template and reactions were carried out using the QuikChange (Agilent, Palo Alto, CA, USA) Site‐Directed Mutagenesis Kit. For introducing the H670A mutation, the following primer pair was used: 5′‐CTGTCTCAAGCTGAAGCTGGTATTGATTCCCAG‐3′ and 5′‐CTGGGAATCAATACCAGCTTC AGCTTGAGACAG‐3′. For mutating H737 to alanine, the following primer pair was used: 5′‐CAGTACGCTCCTGAAGCCTTGATCCTGCAAATC‐3′ and 5′‐GATTTGCAGGATCAAGGCTT CAGGAGCGTACTG‐3′. The PCR product was digested with *DpnI* followed by transformation in *E. coli*. H670A and H737A mutations were confirmed by DNA sequencing. Constructs were then transformed into *GS115* and screened on YPD + Zeocin plates. Expression of Myc*‐*tagged His4 was confirmed by western blotting using anti‐Myc epitope antibodies and the phenotype was examined by plating on YNBD (His^‐^) plates.

The yeast strains used in this study are listed in Table 1.

**Table 1:** List of *K. phaffii* strains used in this study.

Strain	Description	References
*GS115*	*his4*	[39]
*X33*	*HIS4*	Invitrogen
*GS115‐HIS4*	*GS115, HIS4*	This study
*GS115‐HIS4^Myc^*	*GS115, Zeo^r^(P_GAPDH_‐HIS4‐Myc)*	This study
*GS115‐H670A^Myc^*	*GS115, Zeo^r^(P_GAPDH_‐HIS4H670A‐Myc)*	This study
*GS115‐H737A^Myc^*	*GS115, Zeo^r^(P_GAPDH_‐HIS4H737A‐Myc)*	This study
*JC239*	*met2*	[10]
*BY4741*	*MATa his3Δ1 leu2Δ0 met15Δ0 ura3Δ0*	[40]

## Results

### *HIS4* is essential for optimal growth of *K. phaffii* cultured in YPA

**Fig. 1:**
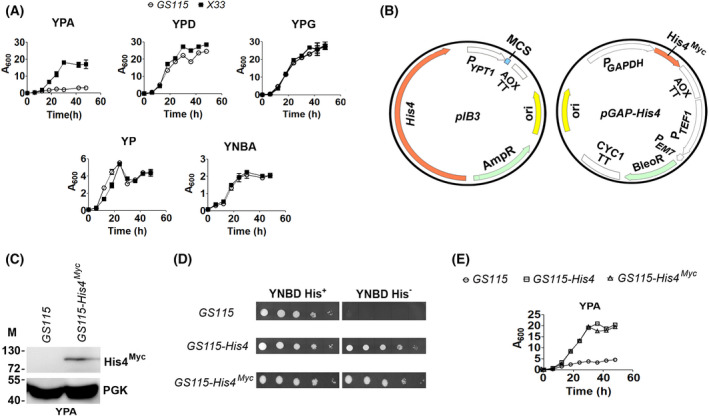
Analysis of the growth of *K. phaffii* prototroph and histidine auxotrophs in YPA. (A) Analysis of growth of *K. phaffii GS115* and *X33* in different media, as indicated. Error bars in each figure indicate SD, *n* = 3. YNB with amino acids was used for the preparation of YNBA. Histidine concentration in YNBA is 20 mg·L^−1^ or 130 µm. (B) Map of pIB3 and pGAP‐His4 vectors. In pIB3 vector (# 25452, Addgene), *K. phaffii HIS4* is expressed from its own promoter. In pGAP‐His4 vector, *HIS4* was amplified from pIB3 with a C‐terminal Myc tag and cloned downstream of the glycerladehyde‐3‐phosphate dehydrogenase (GAP) promoter of *pGAPZA* (ThermoFisher). AOX, alcohol oxidase; GAP, TT, transcription termination sequence; ori, origin of replication; CYC1, cytochrome c1; P_TEF1_, promoter of transcription elongation factor 1; P_EM7_, EM7 promoter for the expression of bleomycin/zeocin resistance gene (BleoR) in *E. coli* and *K*. *phaffii*. Plasmid maps were drawn using SnapGene software (from Insightful Science; available at snapgene.com). (C) Western blot analysis of lysates of *K*. *phaffii* cultured in YPD using Anti‐Myc antibodies. M, molecular weight markers (kDa). (D) Spot assay of different *K. phaffii* strains as indicated in histidine‐sufficient (His^+^) and ‐deficient (His^‐^) YNBD media. (E) Growth curves of different *K. phaffii* strains cultured in YPA, as indicated (*n* = 3).

*K. phaffii* has emerged as an important host for the expression of several heterologous proteins and a model organism for fundamental research [14, 15]. *K. phaffii* expression vectors generally contain auxotroph markers (e.g., *HIS4, MET2, ADE1, ARG4*) or antibiotic selectable markers (e.g., Zeocin, geneticin (G418) and blasticidin S). Expression vectors containing auxotrophic markers such as *HIS4* and *MET2* are transformed into the auxotrophs *GS115* (*his4*) and *JC239* (met2), respectively, while vectors conferring resistance to drugs are transformed into the prototrophic strains such as *X33* [12, 16]. If *GS115* is cultured in minimal media, histidine needs to be supplemented for growth. However, histidine supplementation is not required when *GS115* is cultured in nutrient‐rich media containing 1% yeast extract and 2% peptone (YP) and an appropriate source of carbon. In general, auxotrophic yeast strains defective in amino acid biosynthesis do not exhibit growth defects when cultured in nutrient‐rich media. An exception to this rule was observed when the histidine auxotroph *GS115* exhibited a growth defect when cultured in YP containing 2% acetate (YPA) but not YP, YP containing 2% glucose (YPD) or 2% glycerol (YPG), as well as YNB containing 2% acetate and 130 µm histidine (YNBA) (Fig. 1A). This differential growth of histidine auxotroph and prototroph could be attributed to the selection marker *HIS4,* as both the strains differ only at the *HIS4* locus.

To examine the role of *HIS4* in the growth of *GS115* in YPA, *pIB3* and *pGAP‐His4* vectors were transformed into *GS115*. *pIB3* is a promoter‐less *K. phaffii* vector containing *HIS4* as a selection marker, while *pGAP‐His4* was generated by cloning *HIS4* with a C‐terminal Myc tag (*His4^Myc^*) downstream of *GAPDH* promoter of the *pGAPZA* vector (Fig. 1B). Expression of His4^Myc^ in the *GS115‐His4^Myc^* strain was confirmed by western blotting with anti‐Myc tag antibodies (Fig. 1C). The phenotype of the various strains was examined by spot assay. As expected, *GS115* exhibited histidine auxotrophy, while *GS115‐His4* and *GS115‐His4^Myc^* became prototrophs (Fig. 1D). To assess the effect of *HIS4* expression on the growth of *GS115* in YPA, *GS115*, *GS115‐His4,* and *GS115‐His4^Myc^* were cultured in YPA. The results indicate that, like *X33, GS115‐His4* and *GS115‐His4^Myc^* grew better than *GS115,* suggesting that expression of *HIS4* either from its promoter or from the *GAPDH* promoter reversed the growth defect of *GS115* cultured in YPA (Fig. 1E), indicating that histidine biosynthesis might be essential for the growth of *K. phaffii* in YPA.

### Histidinol dehydrogenase activity of *HIS4* is essential for the growth of *K. phaffii* in YPA

**Fig. 2:**
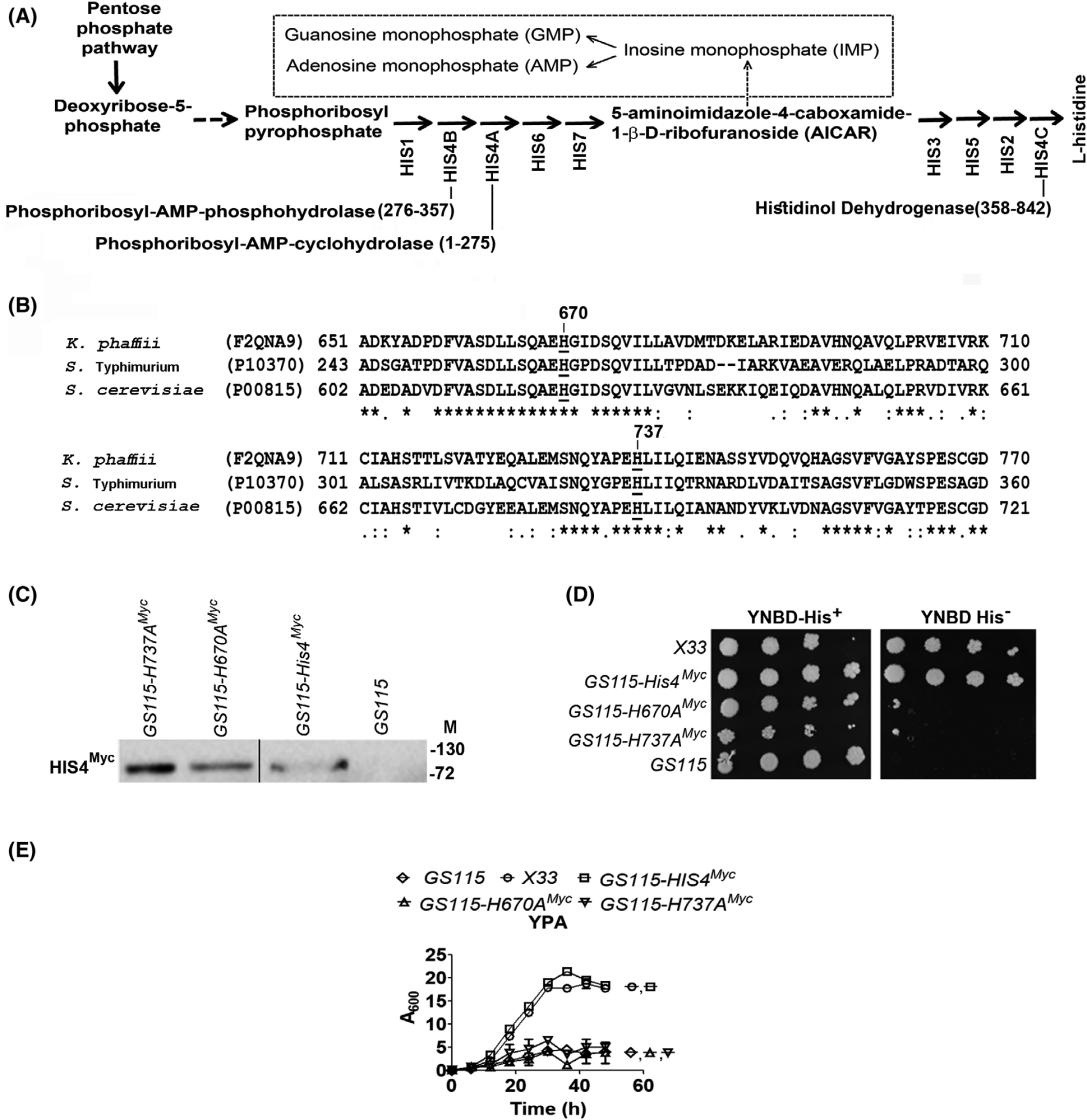
Analysis of the role of *HIS4* in the growth of *K. phaffii* in YPA. (A) Predicted histidine biosynthetic pathway of *K. phaffii*. Numbers in parentheses indicate amino acid residues of the trifunctional enzyme, HIS4 contributing to three different enzymatic activities (HIS4A, HIS4B, HIS4C). (B) Alignment of amino acid sequences of HIS4 of *K. phaffii*, *S. typhimurium,* and *S. cerevisiae*. Histidine residues essential for HIS4C activity are underlined. UniProt IDs are shown in parentheses. Numbers indicate amino acid residues. (C) Western blot analysis of lysates of *GS115* expressing Myc‐tagged HIS4 and HIS4 mutants using anti‐Myc tag antibodies. M, molecular weight markers (kDa). (D) Spot assay of various *K. phaffii* strains as indicated in histidine‐sufficient (His^+^) and ‐deficient (His^‐^) YNBD media. (E) Growth curves of different *K. phaffii* strains cultured in YPA, as indicated. Error bars in each figure indicate SD (*n* = 3).

His4 is a trifunctional protein, catalyzing second, third, and the last reactions of the histidine biosynthetic pathway (Fig. 2A), with amino acids 1–275 encoding for phosphoribosyl‐AMP‐cyclohydrolase activity (His4A), 276–357 encoding for phosphoribosyl‐AMP‐phosphohydrolase activity (His4B), and residues 358–843 coding for the histidinol dehydrogenase activity (His4C) [17]. It is worth mentioning that the histidine biosynthetic pathway is involved not only in histidine production but also in the generation of 5‐aminoimidazole‐4‐carboxamide ribonucleotide (AICAR), which is an essential precursor for purine biosynthesis (Fig 2A). To understand whether His4 is affecting the growth of *GS115* in YPA by modulating histidine production or purine generation, the histidinol dehydrogenase activity of His4 was specifically abrogated. The catalytically active residues of histidinol dehydrogenase have been experimentally characterized in *Salmonella typhimurium* as His261 and His326 [18, 19, 20] and by sequence alignment, the corresponding residues of His4 in *K. phaffii* are identified as H670 and H737 (Fig. 2B). We mutated H670 and H737 to alanine, expressed them as Myc tagged proteins in *GS115,* and confirmed their expression by western blotting using anti‐Myc antibodies (Fig. 2C). *GS115* expressing mutant enzymes exhibited histidine auxotrophy when cultured in YNBD (His^‐^) agar plates, as expected (Fig. 2D). The growth kinetics of *GS115, X33, GS115‐His4^Myc^, GS115‐H670^Myc^, GS115‐H737^Myc^* in YPA medium indicates that strains carrying only the wildtype His4 but not the mutant enzymes restored growth of *GS115* in YPA (Fig. 2E), indicating that His4C, catalyzing the conversion of histidinol to histidine, is essential for the growth of *K. phaffii* in YPA.

### Supplementation of YPA with histidine reverses the growth defect of *K. phaffii GS115*

**Fig. 3:**
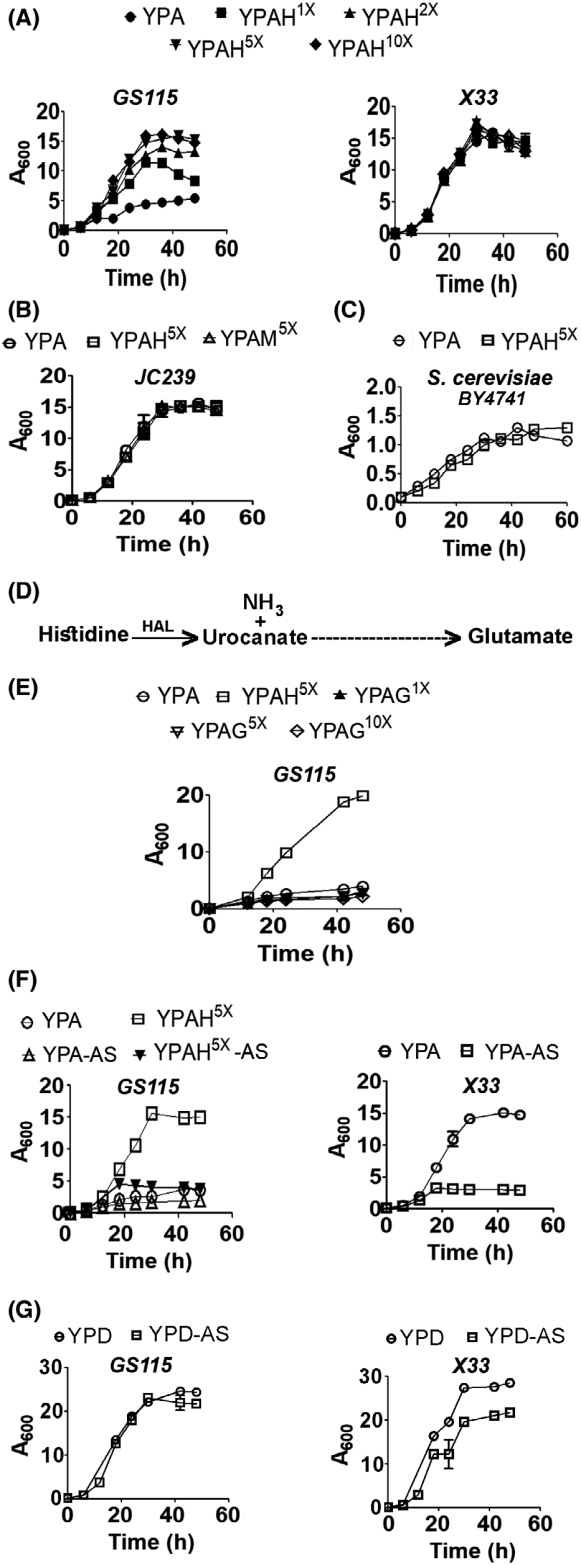
Effect of supplementation of amino acids alone or amino acids and ammonium sulphate on the growth of *K. phaffii* and *S. cerevisiae* cultured in YPA and YPD. (A–C) Growth curves of *K. phaffii GS115, X33,* and *JC239,* as well as *S. cerevisiae BY4741* strains in YPA supplemented with different concentrations of histidine (YPAH) or methionine (YPAM). 1x histidine/methionine is 130 µm (20 mg·L^−1^). Error bars in each figure indicate SD (*n* = 3). (D) Schematic representation of histidine catabolism by the histidine utilization pathway [21, 22]. HAL, Histidine ammonia lyase. (E) Growth curve of *K. phaffii GS115* in YPA supplemented with different concentrations of glutamate (YPAG). 1x glutamate is 130 µm (20 mg·L^−1^). (F and G) Growth curves of *K. phaffii GS115* and *X33* strains cultured in YPA, YPAH5x, or YPD media in the presence or absence of 0.5% ammonium sulphate. Error bars in each figure indicate SD (*n* = 3).

The results thus far indicate that conversion of histidinol to histidine by His4C is essential for the growth of *K. phaffii* in YPA. This led us to investigate the effect of supplementation of YPA with histidine on the growth of *GS115*. *GS115* is generally cultured in shake flasks in minimal media supplemented with 20 mg·L^−1^ or 130 µm of histidine, and hence 130 µm histidine is referred to as 1x in this study. To study the effect of histidine on the growth of *K. phaffii, GS115* and *X33* were cultured in YPA supplemented with 1x (YPAH^1x^), 2x (YPAH^2x^), 5x (YPAH^5x^), or 10x (YPAH^10x^) histidine, and their growth rates were compared. Histidine addition resulted in a significant increase in the growth rate of *GS115,* and peak growth rates were achieved at 5x concentration (Fig. 3A). Hence, YPAH^5x^ was used for further studies. It should be noted that histidine supplementation of YPA enhanced the growth of *GS115* but not *X33* (Fig. 3A).

Next, we assessed the growth of *JC239*, a methionine auxotroph, in the absence and presence of excess methionine or histidine in YPA. The results, however, indicate that the addition of 5x methionine or 5x histidine to YPA did not affect the growth of *JC239* (Fig. 3B). Further, *S. cerevisiae BY4741*, a histidine auxotroph, was cultured in YPA and YPAH^5x^. The results indicate that histidine does not affect the growth of *S. cerevisiae BY4741* (Fig. 3C), suggesting that histidine‐mediated growth is a unique property of *K. phaffii* histidine auxotroph.

Histidine is metabolized to urocanate and ammonia and subsequently to glutamate and formate in bacteria and mammals via the histidine utilization pathway [21, 22] (Fig. 3D). It is possible that a metabolite derived from histidine such as urocanate rather than histidine per se facilitates the growth of *K. phaffii* cultured in YPA. The *K. phaffii* genome does not encode histidine ammonia‐lyase, which catalyzes the conversion of histidine to urocanate. However, histidine may be converted to glutamate by a yet‐to‐be‐discovered pathway in *K. phaffii*. Glutamate, thus generated from histidine catabolism, can enter the TCA cycle via its conversion to α‐ketoglutarate catalyzed by glutamate dehydrogenase and contributes to ATP generation, gluconeogenesis, and growth. If this were to be accurate, glutamate should enhance the growth of *GS115* as efficiently as histidine. However, growth of *GS115* was not enhanced when cultured in YPA containing 5x (YPAG^5x^) or 10x glutamate (YPAG^10x^) (Fig. 3E), suggesting that glutamate derived from histidine is unlikely to be the growth promoter of *GS115*. To examine whether ammonium derived from histidine catabolism is contributing to growth, we cultured *K. phaffii* cultured in YPA supplemented with ammonium sulfate (AS). Surprisingly, AS inhibited the growth of *GS115* in YPAH^5x^ as well as *X33* in YPA (Fig. 3F), but not in YPD (Fig. 3G). Taken together, these results indicate that neither glutamate nor ammonium derived from histidine catabolism is contributing to the growth of *K. phaffii* metabolizing acetate in the presence of yeast extract and peptone.

### Identification of histidine responsive genes of *K. phaffii*

**Fig. 4:**
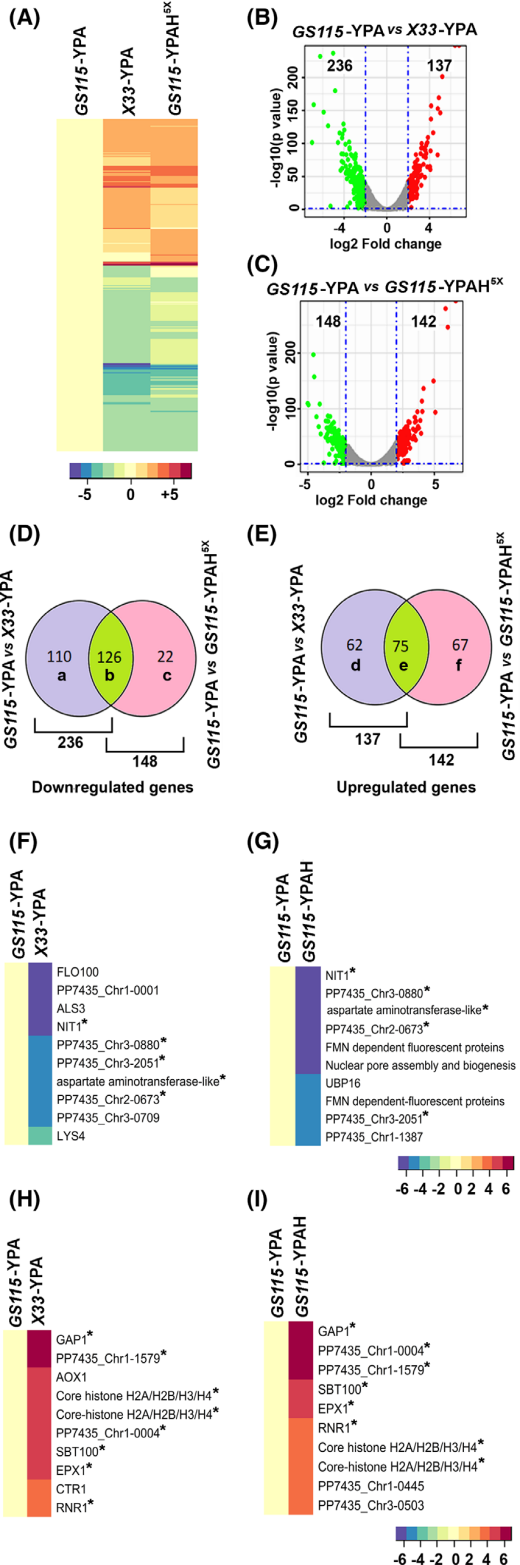
Effect of histidine on *K. phaffii* transcriptome. (A–C) Heat map and Volcano plots depicting overall change in the mRNA profile in the presence of histidine. (D,E) Venn diagrams representing the number of genes in each sector. b and e depict shared differentially expressed genes in both the test conditions, whereas a, c, d, f depict exclusive down‐ or upregulated genes. (F,G) Topmost downregulated genes are presented in heat maps. (H,I) Topmost upregulated genes are presented in heat maps. Asterisks depict the shared genes in histidine synthesized vs. histidine‐ supplemented conditions. Even though *GS115/X‐33* was used for RNAseq, gene names are derived from *CBS7435,* since the CBS7435 genome sequence has been analyzed more carefully and annotated manually.

In the present study, growth promotion of *K. phaffii* was observed not only by histidine added to the culture medium but also by histidine biosynthesized intracellularly, thus ruling out the involvement of signaling pathways mediated by cell surface proteins. We reasoned that histidine‐mediated growth of *K. phaffii* cultured in YPA may involve the regulation of expression of genes via a yet‐to‐be‐discovered intracellular signaling pathway. Since signal transduction pathways ultimately culminate in the regulation of gene expression, we examined whether intracellular biosynthesis of histidine or histidine acquired from the culture medium can alter the gene expression profile of *K. phaffii*. A genome‐wide, high‐throughput RNA‐Seq was carried out with RNA isolated from *GS115* and *X33* cultured in YPA as well as *GS115* cultured in YPAH^5x^ (Table S1). The raw data files have been submitted to the GEO database with an accession number GSE174633. *GS115* cultured in YPA (*GS115*‐YPA) was used as a control for comparing the transcriptomes, and a *P‐*value <0.05 and log2‐fold change of ±2 was set as the threshold. The results indicate that histidine taken up by *GS115* cultured in YPA (*GS115*‐YPAH^5x^) or synthesized intracellularly in *X33* (*X33*‐YPA) alters the transcriptome of *K. phaffii* (Fig. 4A–C).

**Fig. 5:**
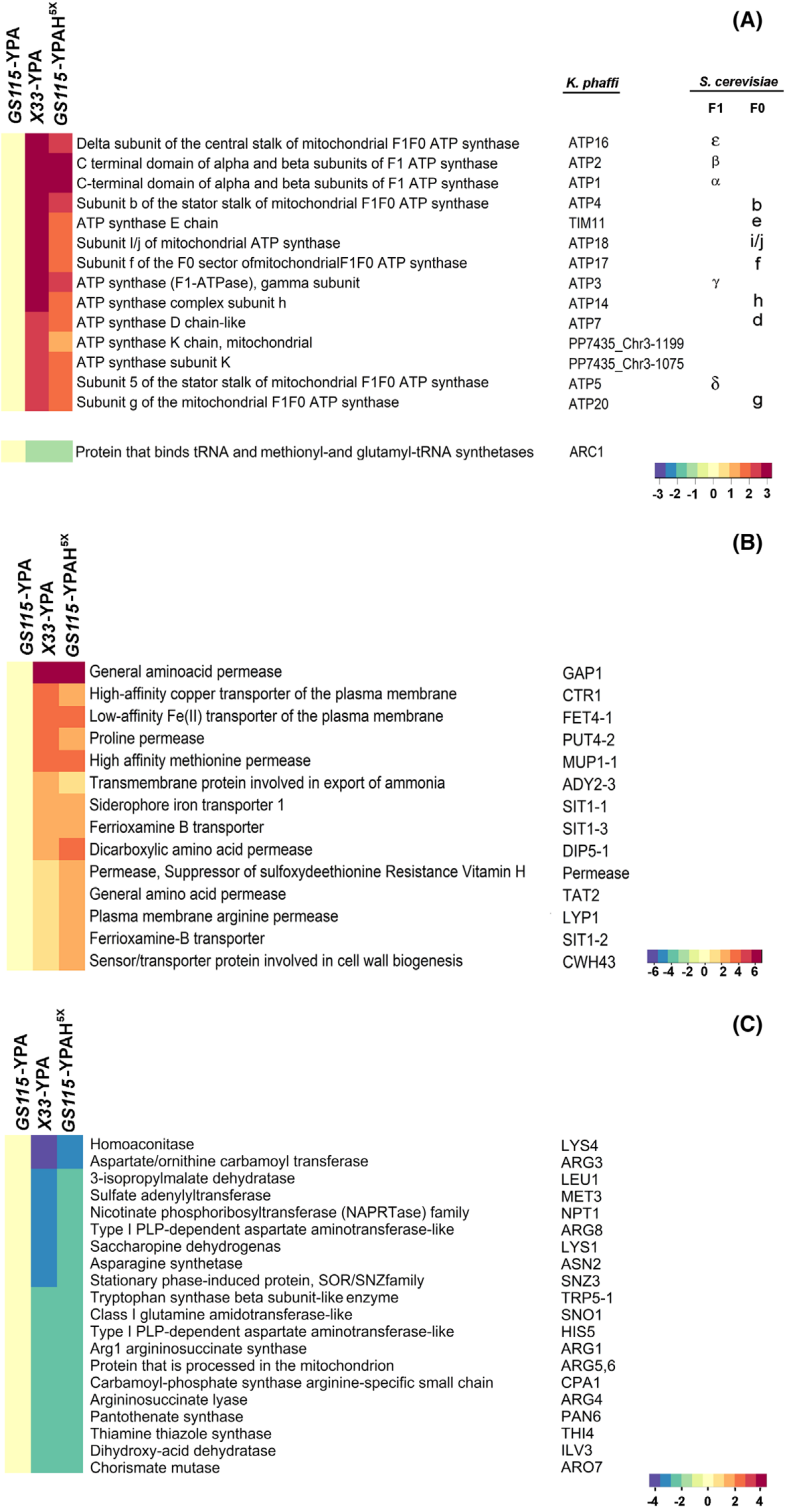
Identification of histidine‐responsive gene groups. (A) Genes ≥2‐fold upregulated in either *X33*‐YPA or *GS115*‐YPAH^5x^ encoding for ATP synthase subunits. Bottom panel depicts ~1‐‐fold downregulation of gene involved in repression of ATP synthase subunit expression, *ARC1*. (B) Genes ≥2‐fold upregulated in either *X33*‐YPA or *GS115*‐YPAH^5x^ encoding for transporters and nutrient uptake permeases (C). Genes ≤ −2 downregulated in either *X33*‐YPA or *GS115*‐YPAH^5x^ encoding for enzymes for amino acid and micronutrient biosynthesis. Heat map illustrates expression levels of genes, along with its description and annotation. Even though *GS115/X‐33* was used for RNAseq, gene names are derived from *CBS7435* since CBS7435 genome sequence has been analyzed more carefully and annotated manually.

Interestingly, the overall changes in the transcriptome of *X33*‐YPA are similar to that of *GS115*‐YPAH^5x^. Among the 236 and 148 genes downregulated in *X33*‐YPA and *GS115*‐YPAH^5x,^, respectively, 126 are downregulated in both (Fig. 4D). Similarly, among the 137 and 142 genes upregulated in *X33*‐YPA and *GS115*‐YPAH^5x,^, respectively, 75 are upregulated in both (Fig. 4E). Also, five of the ten most downregulated genes and eight among the ten most upregulated genes are shared between *GS115*‐YPAH^5x^ and *X33*‐YPA (Fig. 4F–I). Thus, histidine responsive genes, which are activated or repressed in *K. phaffii* histidine auxotroph (*GS115*) cultured in YPAH (*GS115*‐YPAH^5x^) as well as *K. phaffii* prototroph (*X33*) cultured in YPA (*X33*‐YPA) may have a key role in promoting the growth of *P. pastoris* cultured in YPA. Further analysis of histidine‐responsive genes differentially expressed in both *GS115*‐YPAH^5x^ and *X33*‐YPA (b and e of Fig. 4D,E) revealed that 14 genes described as nuclear‐encoded subunits of mitochondrial F1F0 ATP synthase, involved in oxidative phosphorylation (Fig. 5A, top panel), are upregulated in *GS115*‐YPAH^5x^ as well as *X33*‐YPA. Further, *ARC1,* annotated as a protein that binds tRNA and methionyl‐ and glutamyl‐tRNA synthetases (https://www.uniprot.org/uniprot/P46672), is downregulated in *GS115*‐YPAH^5x^ and *X33*‐YPA. When *ARC1* is downregulated, methionyl‐tRNA synthetase localizes to the nucleus and activates the transcription of several genes involved in oxidative phosphorylation, including those encoding mitochondrial F1F0 ATP synthase [23, 24]. Another set of genes upregulated in both *GS115*‐YPAH^5x^ and *X33*‐YPA include those annotated as general amino acid, proline, methionine, arginine, and dicarboxylic amino acid permeases and transporters such as iron transporter, copper transporter, siderophore ion transporter, and ferrioxamine B transporter (Fig. 5B). Genes that are downregulated in both *GS115*‐YPAH^5x^ and *X33*‐YPA include those encoding enzymes involved in the biosynthesis of amino acids and micronutrients (Fig. 5C). Overall, these results suggest that enhanced oxidative phosphorylation, facilitation of nutrient import, and inhibition of biosynthetic reactions are likely to result in an increase in intracellular ATP levels leading to the robust growth of *K. phaffii* in YPA.

## Discussion

A serendipitous observation that prototrophic (*X33*) and histidine auxotrophic (*GS115*) strains of *K. phaffii* exhibit differential growth when cultured in YPA medium led to the identification of histidine‐responsive genes, hitherto unreported in any yeast species. The nexus between histidine auxotrophy and growth defect in cells cultured under nutrient‐rich conditions is rather puzzling, since yeast auxotrophs rarely exhibit a phenotype when cultured in yeast extract and peptone, a rich source of amino acids and other metabolic intermediates. Thus, defects in amino acid biosynthetic pathways do not affect the growth of auxotrophs under these culture conditions, as observed in the case of *GS115* and *X33* cultured in YPD, YPG, and YP. The fact that histidine auxotrophy affects growth only in cells cultured in YPA motivated us to investigate this phenomenon further. We first demonstrated that *K. phaffii GS115* lacking *HIS4* does not grow as efficiently as *X33* in YPA, and expression of *HIS4* in *GS115* reverses the growth defect. Analysis of mutations that affect *HIS4C* indicated that disruption of histidinol dehydrogenase activity of *HIS4,* required for the conversion of histidinol to histidine, the last step of histidine biosynthesis, is responsible for the growth defect of *GS115*. Thus, disruption of the biosynthesis of histidine but not purine causes a growth defect in *K. phaffii* when cultured in YPA. Subsequent studies indicated that histidine, the endproduct of the histidine biosynthetic pathway, is required for optimal growth of *K. phaffii* in YPA. Histidine‐dependent growth in YPA is unique to *K. phaffii* and is not observed in *S*. *cerevisiae BY4741*, a histidine auxotroph, carrying a deletion in *HIS3*.

Histidine transamination results in the generation of glutamate through the action of ARO8 in *Candida glabrata* [25] whereas it is deaminated to generate glutamate and ammonia in bacteria and mammals [22]. While glutamate can enter the TCA cycle and contribute to ATP generation, ammonia serves as the inorganic nitrogen donor. A role for histidine‐derived glutamate and ammonium in the growth of *K. phaffii* in YPA is unlikely, since neither facilitates growth in YPA. Thus, histidine mediates the growth of *K. phaffii* only in the presence of an organic source of nitrogen, such as amino acids. Whether ammonium sulfate interferes with the regulation of histidine‐responsive genes in cells cultured in YPA remains to be investigated.

Histidine/histidine derivatives are known to have pleiotropic functions in different organisms. For example, imidazole propionate derived from histidine of gut microbiota in individuals with type 2 diabetes functions as a signaling molecule involved in activating mammalian Target of Rapamycin 1 [26]. Histidine is involved in the regulation of glucagon prehormone, and preproglucagon mRNA in the pancreas [27]. Histidine supplementation is used as therapy in multiple diseases due to its antioxidant and antiinflammatory properties, proton‐buffering power, metal ion chelation, protection against glycation, and lipooxidation [28, 29, 30]. In *S. cerevisiae*, histidine maintains copper homeostasis [31] and affects biofilm‐forming Flor yeast's growth by completely inhibiting the biofilm formation by nonspecific interactions [32]. Histidine uptake in a few bacteria has been associated with the maintenance of pH and redox potential. For example, in *Lactobacillus buchneri*, histidine is imported via histidine/histamine antiporter, and once inside the cell, histidine gets decarboxylated to form histamine, which has an additional positive charge. Excretion of this histamine results in a net positive charge in the medium. This proton motive force has been attributed to generate ATP by the F1F0 ATPase [33, 34]. However, this mechanism is unlikely to operate in *K. phaffii,* since its genome does not encode histidine decarboxylase required to synthesize histamine from histidine. Specific aminoacyl tRNA synthetases function as amino acid sensors and regulate cell signaling, apoptosis, or inflammation [35, 36]. Whether histidyl tRNA synthetases can function as histidine sensors and regulate growth in *K. phaffii* is a topic of future study.

The fact that imidazole propionate derived from histidine of gut microbiota in individuals with type 2 diabetes functions as a signaling molecule involved in activating mammalian Target of Rapamycin [26] prompted us to investigate whether histidine can alter gene expression of *P. pastoris* cultured in YPA by RNA seq. We have identified several genes whose expression is modulated by histidine synthesized intracellularly as well as that obtained extracellularly. High similarity in the gene expression profiles of *GS115*‐YPAH^5x^ and *X33*‐YPA suggests that histidine regulates gene expression after entry into the cells via an intracellular signal transduction pathway rather than via cell surface proteins. The fact that several genes involved in mitochondrial oxidative phosphorylation are upregulated in *GS115*‐YPAH^5x^ and *X33*‐YPA suggests that enhanced production of ATP may be one of the mechanisms by which histidine promotes growth. In *S. cerevisiae*, high acetate levels result in rapid ATP depletion due to its conversion to Acetyl‐Co‐A, and as a result cells enter into a prolonged lag phase and normal growth is restored only after several days [38]. Rapid depletion of ATP does not occur in cells cultured in YP or YPG, since acetate production and its conversion to Acetyl‐Co‐A is not high enough to result in ATP depletion. Thus, histidine‐mediated activation of transcription of genes required for ATP synthesis in *GS115*‐YPAH^5x^ and *X33*‐YPA YPA may result in rapid replenishment of ATP, leading to normal growth. It is tempting to speculate that low ATP levels in *K. phaffii* cultured in YPA is sensed by a histidine‐mediated signaling pathway leading to upregulation of nuclear genes encoding F1F0ATPase, enhanced oxidative phosphorylation, restoration of intracellular ATP levels, and growth. Since metabolic intermediates such as amino and ketoacids are readily available in the YPA medium, the newly synthesized ATP is used for cell division and growth rather than biosynthetic processes. The fact that genes encoding several permeases and transporters are also upregulated in *GS115*‐YPAH^5x^ and *X33*‐YPA suggests that biosynthetic intermediates can be rapidly imported into the cell, thus favoring growth. In this context, it is pertinent to note that *GAP1* encoding the general amino acid permease involved in the transport of several amino acids [37] is also highly upregulated in *GS115*‐YPAH^5x^ and *X33*‐YPA (Fig. 4H). It is possible that histidine has to compete with other amino acids and peptides in YPA for its import via GAP. One way to improve the efficiency of histidine import is by increasing the amount of histidine in the medium. Thus, histidine may be imported more efficiently in *GS115* cultured in YPAH^5x^ than YPAH^1x^.

Histidine is known to act as a buffer and it is possible that the buffering property of histidine added to the culture medium may enhance nutrient uptake and contribute to growth. However, this is unlikely, since histidine biosynthesized intracellularly also promotes growth of *K. phaffii* in YPA. It is pertinent to note that lysates of *GS115* and *X33* cultured in YPA, when examined by SDS‐PAGE followed by Coomassie Brilliant Blue staining, exhibit similar protein profiles (data not shown), suggesting that histidine auxotrophy does not result in the deficiency of Coomassie blue‐stainable proteins in these lysates. However, it is possible that histidine deficiency results in a decrease in the synthesis of certain histidine‐rich proteins essential for the growth of cells cultured in YPA which are less abundant and hence cannot be visualized in SDS polyacrylamide gels.

Yeast extract contains B‐complex vitamins and amino acids necessary for growth, while peptone acts as the source of nitrogen, vitamins, and minerals. They are routinely used for culturing recombinant yeast strains producing drugs, vaccines, and biotherapeutics. This study demonstrates for the first time that media containing yeast‐based organic nitrogen sources and acetate as carbon spruce are not preferable for the growth of histidine auxotrophs of *K. phaffii* such as *GS115*. We recommend using *K. phaffii* prototrophs rather than histidine auxotrophs for high‐density fermentation and production of acetate‐derived compounds.

## Conflict of interest

The authors declare that they have no conflicts of interest for the contents of this article.

## Author contributions

AG and PNR conceived and designed the project, analyzed the data, and wrote the article. AG carried out the experiments. PNR obtained funding.

## Supporting information


**Table S1**. RNA seq analysis of GS115 and X33 cultured in YPA and GS115 cultured in YPAH5X.

## Data Availability

The RNA sequencing data are available at NCBI Gene Expression Omnibus (https://www.ncbi.nlm.nih.gov/geo/ under the accession number GSE174633.
